# Tempol Preserves Endothelial Progenitor Cells in Male Mice with Ambient Fine Particulate Matter Exposure

**DOI:** 10.3390/biomedicines10020327

**Published:** 2022-01-29

**Authors:** Xuanyou Liu, Aimin Wang, Zhiheng Chen, Yuqi Cui, Hong Hao, Timothy L. Domeier, Qinghua Sun, Zhenguo Liu

**Affiliations:** 1 Center for Precision Medicine and Division of Cardiovascular Medicine, Department of Medicine, University of Missouri School of Medicine, Columbia, MO 65212, USA; liuxua@health.missouri.edu (X.L.); wangaimin@csu.edu.cn (A.W.); czh15873170803@126.com (Z.C.); cuiyu@health.missouri.edu (Y.C.); haoho@health.missouri.edu (H.H.); 2 Department of Medical Pharmacology and Physiology, University of Missouri School of Medicine, Columbia, MO 65212, USA; domeiert@health.missouri.edu; 3 College of Public Health, the Ohio State University, Columbus, OH 43210, USA; qhsun@yahoo.com

**Keywords:** particulate matter, endothelial progenitor cells, reactive oxygen species, SOD1, Tempol, sex difference

## Abstract

Ambient fine particulate matter (PM) exposure associates with an increased risk of cardiovascular diseases (CVDs). Major sex differences between males and females exist in epidemiology, pathophysiology, and outcome of CVDs. Endothelial progenitor cells (EPCs) play a vital role in the development and progression of CVDs. PM exposure-induced reduction of EPCs is observed in male, not female, mice with increased reactive oxygen species (ROS) production and oxidative stress. The lung is considered an important source of ROS in mice with PM exposure. The aim of the present study was to investigate the sex differences in pulmonary superoxide dismutase (SOD) expression and ROS production, and to test the effect of SOD mimic Tempol on the populations of EPCs in mice with PM exposure. Both male and female C57BL/6 mice (8–10 weeks) were exposed to intranasal PM or vehicle for 6 weeks. Flow cytometry analysis demonstrated that PM exposure significantly decreased the levels of EPCs (CD34^+^/CD133^+^) in both blood and bone marrow with increased ROS production in males, but not in females. ELISA analysis showed higher levels of serum IL-6 and IL-1βin males than in females. Pulmonary expression of the antioxidant enzyme SOD1 was significantly decreased in males after PM exposure, but not in females. Administration of the SOD mimic Tempol in male mice with PM exposure attenuated the production of ROS and inflammatory cytokines, and preserved EPC levels. These data indicated that PM exposure-induced reduction of EPC population in male mice may be due to decreased expression of pulmonary SOD1 in male mice.

## 1. Introduction

Ambient fine particulate matter (PM) is the key component of air pollution. PM is categorized according to aerodynamic diameter into coarse (PM_10_, diameter < 10 μm), fine (PM_2.5_, diameter < 2.5 μm), and ultrafine (PM_0.1_, diameter < 0.1 μm) [1]. Exposure to PM_2.5_ and PM_0.1_ are linked with increased cardiovascular morbidity and mortality as they can penetrate small airways and alveoli [2]. Long- or short-term PM_2.5_ exposure enhances the development and progression of cardiovascular diseases (CVDs), including ischemic heart disease, heart failure, arrhythmias, hypertension, and atherosclerosis [3,4]. Endothelial cell (EC) dysfunction plays a critical role in the pathogenesis of CVDs [5,6]. Endothelial progenitor cells (EPCs) that are primarily derived from bone marrow or tissue-resident cells can restore dysfunctional endothelium and maintain normal cardiovascular function [7]. Abnormalities in the number and function of EPCs are closely related to CVDs including atherosclerosis and myocardial infarction [8,9]. Previous studies have shown that PM exposure reduces the number and function of EPCs in both animals and human subjects [10,11].

PM exposure increases reactive oxygen species (ROS) production and oxidative stress [12,13]. Uncontrolled oxidative stress and excessive ROS production are among the leading causes for a variety of CVDs, such as atherosclerosis, hypertension, cardiomyopathy, and myocardial infarction [14,15]. Our previous study has demonstrated that PM exposure selectively decreases the circulating EPCs population in male mice via increased oxidative stress and apoptosis, but the molecular mechanisms were not clear [16]. Several studies have shown that oxidative stress in the lung is the key initiator for PM exposure-induced reduction of EPCs [12,17]. Superoxide dismutase-1 (SOD1), an antioxidant enzyme which is mainly expressed in the mitochondrial intermembrane space and cytosol, is closely associated with PM exposure-induced generation of ROS primarily from site III of the mitochondrial electron transport chain [18]. Respiratory deposition of PM in the lung leads to pulmonary ROS formation and initiates inflammatory responses and oxidative stress [19]. Preventing PM_2.5_ exposure-induced pulmonary oxidative stress using lung-specific overexpression of ecSOD in mice has been shown to restore EPC levels in blood and bone marrow [12]. However, it is unclear if there is a significant difference in SOD expression in the lung between males and females with PM exposure.

Tempol (4-hydroxy-2,2,6,6-tetramethylpiperidine-1-oxyl) is a SOD mimic that catalyzes superoxide anion (O2.^−^) to hydrogen peroxide (H_2_O_2_) [20]. Tempol has been shown to attenuate ROS generation, improve cardiac function and insulin sensitivity, and decrease blood pressure [21,22,23]. Treatment of spontaneous hypertensive rats with Tempol increases the number of circulating EPCs in association with decreased levels of oxidation [24]. Tempol also protects mouse lungs from oxidative damage and inflammation induced by cigarette smoke exposure [25]. However, it is unclear if Tempol could prevent PM exposure-induced reduction of EPCs. The present study was designed to test the hypothesis that pulmonary SOD expression was selectively decreased in males in association with increased ROS production and decreased EPC levels. The objectives were: (1) to investigate the sex differences in pulmonary SOD expression in response to PM exposure in mice; and (2) to determine if treatment with the SOD mimic Tempol could prevent PM exposure-induced ROS production and reduction of EPCs in mice.

## 2. Materials and Methods

### 2.1. PM Exposure and Animal Model

The animal study was performed according to the “Guide for the Care and Use of Laboratory Animals of the US National Institutes of Health.” All animal protocols and experiments were reviewed and approved by the Animal Care and Usage Committee of the University of Missouri-Columbia, MO, USA (#9227, 9 May 2018). Both male and female wild-type C57 BL/6 mice (8–10 weeks old, from the Jackson Laboratory, Bar Harbor, ME, USA) were randomly divided into control and PM exposure groups. Different sources of PM contain different components with different sizes. The composition of PM is a mixture of various particles including metals, crustal materials, and bio-aerosols [16,26]. Since the deleterious effects on cardiovascular system related to PM exposure is predominantly induced by PM_2.5_, the PM preparation of <4 μm (Standard Reference Materials 2786 from The National Institute of Standards and Technology) was used in the present study to ensure consistency and reproducibility as described in our previous study [16]. PM particles were prepared in endotoxin-free PBS with a concentration of 0.5 µg/μL. Mice were anesthetized with 2% isoflurane and exposed to 10 µg PM three times per week for 6 weeks via intranasal instillation, with endotoxin-free PBS as control as described [10,16]. To decrease the ROS level, mice were pre-treated with SOD mimic Tempol (1 mM in drinking water, Sigma, Saint Louis, MO, USA) for 24 h prior to PM exposure with continuation of Tempol treatment for the rest of experiment as described [27]. This mouse model with Tempol in drinking water has been well established with the plasma Tempol concentration of 6 to 7 μmol/L [27,28,29].

### 2.2. Flow Cytometry Analysis for EPCs, Intracellular ROS Level and Cell Apoptosis

Blood cells and bone marrow cells were collected and prepared from mice after 6 weeks of PM or vehicle exposure for analysis of EPCs, intracellular ROS level and apoptosis following the removal of red blood cells (RBC) using RBC lysis buffer as described [10,16]. For EPC analysis, CD34^+^/CD133^+^ cell population was determined using flow cytometry as described [11,16,30]. The antibodies of CD34^+^ AF700 and CD133^+^ APC were purchased from BioLegend (San Diego, CA, USA). Intracellular ROS level in CD34^+^/CD133^+^ cells were quantitatively measured using FITC-ROS detection reagents (Invitrogen) as described [16,31]. The cells were incubated with the reagent at 37 °C for 10 min. After 2 times of washing with PBS, the labeled cells were suspended in warm PBS and analyzed with flow cytometry. The apoptotic rate of EPCs was determined using the FITC Annexin V apoptosis detection kit from BD (Cat#556547) as per the manufacturer’s protocol. The fluorescence-positive cells were quantitatively evaluated using a Flow Cytometer LSR II (BD Bioscience, San Jose, CA, USA) and software FlowJo_V10.

### 2.3. Measurement of Inflammatory Cytokines

Mouse blood samples were obtained after 6 weeks of PM or vehicle exposure. Serum was prepared with centrifuging the blood samples for 20 min at 300 g. The pro-inflammatory cytokines interleukin (IL)-1β (Cat#432604), and IL-6 (Cat#431304) were measured with a mouse cytokine 32-plex discovery assay by Eve Technologies, Corp. (Calgary, AB, Canada) or with an ELISA kit (BioLegend, San Diego, CA, USA).

### 2.4. Western Blot

Mouse lung tissue was collected after 6 weeks of PM exposure. The protein was extracted for Western Blot analysis. SOD1 primary antibody (1:800, ThermoFisher, Berkeley, MO, USA, Cat # MA1-105), β-actin (1:2000, Santa Cruz, Dallas, TX, USA, #sc-47778 HRP), and corresponding secondary antibodies (1:2000, CST, Cat #7076) were incubated with the protein preparations according to the manufacturer’s recommendation. Immunoreactive bands were visualized using chemiluminescence (ECL Kit; Pierce Biotechnology, Waltham, MA, USA) and captured with a LI-COR molecular imager. The total intensity of the band for each protein was calculated with Image Studio from LI-COR Biosciences and normalized to that of β-actin.

### 2.5. Statistical Analysis

All data were presented as mean ± standard error of the mean (SEM) and analyzed using GraphPad Prism 8.4.2. (GraphPad, San Diego, CA, USA) and SPSS Statistics 18.0 (IBM Corp., Armonk, NY, USA). One-way ANOVA (analysis of variance) with Tukey’s post hoc analysis was used to analyze the data. A *p* value of <0.05 was considered statistically significant.

## 3. Results

### 3.1. Levels of Circulating and Bone Marrow EPCs Were Decreased with Increased Apoptosis in Male Mice with PM Exposure

**Figure 1:**
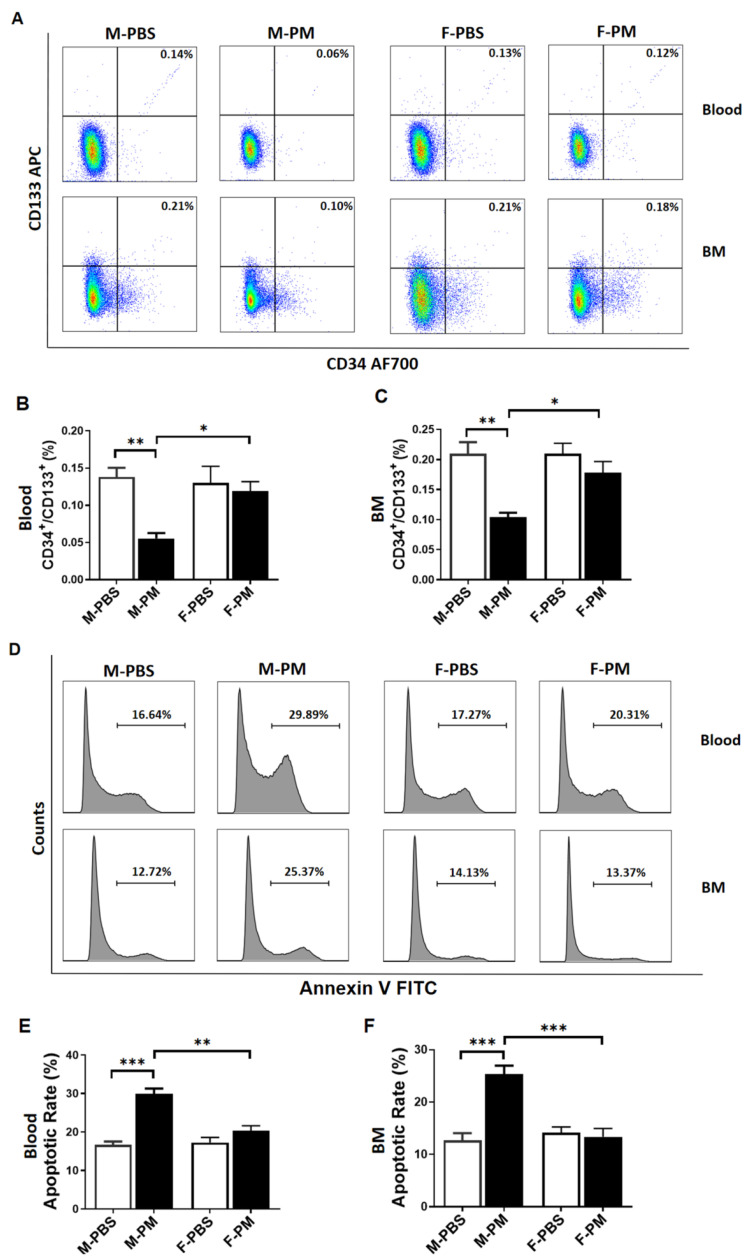
**PM exposure selectively decreased EPC levels in blood and bone marrow with an increased apoptosis rate in male mice**. (**A**) White blood cells were stained with CD133 APC and CD34 AF700 antibodies for flow-cytometric analysis of circulating (**upper** panel) or bone marrow (**lower** panel) EPCs (CD34^+^/CD133^+^), with summary data (**B**,**C**) demonstrating that PM exposure selectively decreased the EPCs populations in blood and bone marrow in male mice (*n* = 6). (**D**) Cells’ apoptotic rates of EPCs in blood and bone marrow as determined by flow cytometry analysis of Annexin V-FITC stained cells, with summary data for EPCs in blood (**E**) and in bone marrow (**F**) showing that PM exposure selectively increased EPCs apoptotic rates in male, but not in female, mice (*n* = 6). M-PBS: male mice with PBS treatment; M-PM: male mice with PM exposure; F-PBS: female mice with PBS treatment; F-PM: female mice with PM exposure. BM: bone marrow. * *p* < 0.05, ** *p* < 0.01 and *** *p* < 0.001.

EPCs in blood and bone marrow are important sources of EPCs [7,10,32]. Cells double positive for CD34 and CD133 (CD34^+/^CD133^+^) have been widely accepted as EPCs [11,16,30]. Flow cytometry analysis was used to determine the levels of EPCs in blood and bone marrow in mice following 6 weeks of PM exposure. As shown in Figure 1A, PM exposure significantly decreased the number of CD34^+^/CD133^+^ cells in male mice both in blood and bone marrow. However, no significant changes in EPC populations were observed in female mice with PM exposure (Figure 1A–C). The apoptosis rate of CD34^+/^CD133^+^ cells in blood and bone marrow was significantly increased in male mice with PM exposure, as compared to the control group, but not in female mice (Figure 1D–F). These results indicate that decreased numbers of EPCs in the circulation and in bone marrow in male mice with PM exposure might be due to an increased apoptosis.

### 3.2. Cytokines and ROS Levels Were Significantly Increased in Male Mice with PM Exposure with Decreased Pulmonary SOD1 Expression

**Figure 2:**
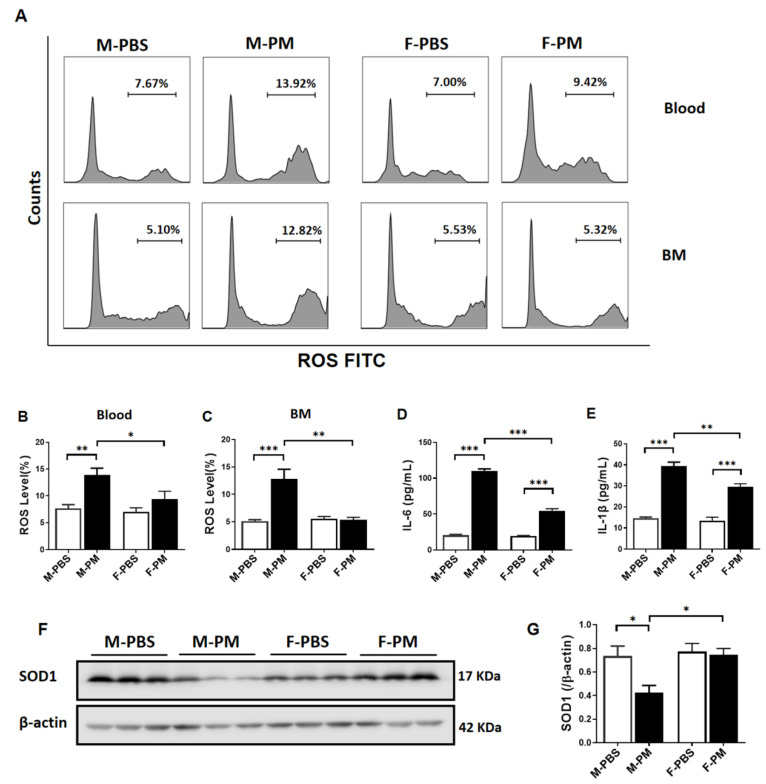
**PM exposure decreased lung SOD1 expression with increased serum cytokines and intracellular ROS production**. (**A**) ROS production in CD34^+^/CD133^+^ cells was analyzed using flow cytometry, with summary data showing a significant increase in intracellular ROS production in male mice with PM exposure compared to the control group and female mice with PM exposure in blood (**B**) and bone marrow (**C**) (*n* = 6). Serum IL-6 (**D**) and IL-1β (**E**) were significantly increased in both male and female mice with PM exposure compared to the PBS control, while their concentrations in male mice were significantly higher than in female mice (*n* = 5). (**F**) Protein expression of SOD1 in the lung, with summary data (**G**) showed significantly decreased SOD1 in male mice but not in female mice (*n* = 3). The experiment was repeated three times independently. M-PBS: male mice with PBS treatment; M-PM: male mice with PM exposure; F-PBS: female mice with PBS treatment; F-PM: female mice with PM exposure. BM: bone marrow. * *p* < 0.05, ** *p* < 0.01 and *** *p* < 0.001.

Intracellular ROS level in CD34^+^/CD133^+^ cells in both blood and bone marrow was significantly increased in males, but not in females, with PM exposure (Figure 2A–C). Serum levels of IL-6 and IL-1β were increased both in male and female mice following PM exposure (Figure 2D,E). However, PM exposure-induced production of IL-6 and IL-1β were significantly higher in males (IL-6, 110.3 ± 2.78 pg/mL; IL-1β, 39.37 ± 1.95 pg/mL) than that in females (IL-6, 54.59 ± 2.87 pg/mL; IL-1β, 29.54 ± 1.47 pg/mL) (Figure 2D,E).

To determine if there was a sex difference in pulmonary SOD1 expression in mice with PM exposure, lung tissues were collected after 6 weeks of PM exposure. Western blotting analysis showed that the protein level of SOD1 was substantially decreased in male mice exposed to PM, as compared to the control, while no significant change in SOD1 expression was observed in female mice with PM exposure (Figure 2F,G).

### 3.3. Treatment with SOD Mimic Tempol Prevented PM Exposure-Induced Production of Cytokines and ROS and Reduction of EPCs in Male Mice

**Figure 3:**
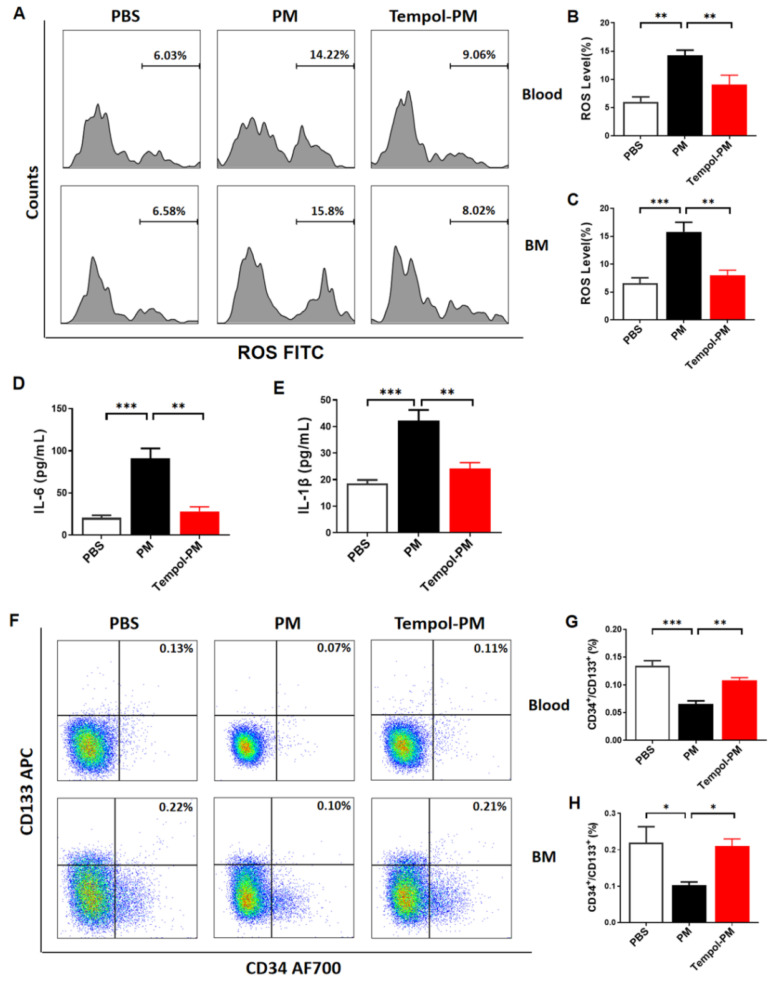
**Tempol treatment preserved the EPCs in male mice with PM exposure**. (**A**) Flow cytometry analysis was used to determine the ROS level in CD34^+^/CD133^+^ cells, with summary data showing that increased ROS production exposed to PM was blocked by Tempol treatment in the blood (**B**) and bone marrow (**C**). Elevated serum levels of pro-inflammatory cytokines IL-6 (**D**) and IL-1β (**E**) induced by PM were effectively prevented by Tempol treatment. (**F**) Flow cytometry analysis for circulating EPCs (CD34^+^/CD133^+^), with summary data showing that the PM exposure-induced decrease in the circulating EPC level in male mice was restored after Tempol treatment in the blood (**G**) and bone marrow (**H**). *n* = 5. PBS: male mice with PBS treatment; PM: male mice with PM exposure; Tempol-PM: male mice with PM exposure and Tempol treatment. BM: bone marrow. * *p* < 0.05, ** *p* < 0.01, and *** *p* < 0.001.

To determine the role of SOD in the PM exposure-induced decrease of EPCs, male mice were treated with SOD mimic Tempol. Indeed, PM exposure-induced elevation of intracellular ROS was effectively attenuated with Tempol treatment (Figure 3A–C). In parallel with the decreased intracellular ROS production, no significant increase of serum levels of IL-6 and IL-1β were observed in mice co-treated with PM and Tempol (Figure 3D,E). Tempol treatment also effectively restored the levels of EPCs both in blood and bone marrow in male mice with PM exposure (Figure 3F–H). Since there were no significant changes in intracellular ROS and populations of EPCs in the bone marrow and blood in female mice with PM exposure, female mice were not treated with Tempol in the present study.

## 4. Discussion

The number and function of EPCs are closely associated with the development and progression of CVDs [33]. Decreased number or dysfunction of EPCs leads to impaired vascular integrity and angiogenesis [33,34]. PM exposure increases the risk of CVDs and decreases the number and function of EPCs [30,35]. We have previously shown that EPCs’ population in blood is decreased in male, but not in female, mice following PM exposure, due to increased ROS production. Furthermore, the sex differences were found to be independent of the female sex hormone estrogen [16]. In the present study, we demonstrated that both circulating EPC and bone marrow EPC levels were significantly decreased in male mice, but not in females, following PM exposure. PM exposure in male, but not female, mice also increased production of pro-inflammatory cytokines and intracellular ROS, with decreased protein expression of the antioxidant enzyme SOD1 in the lung. SOD mimic Tempol treatment effectively prevented PM exposure-induced intracellular ROS production and increase of serum IL-6 and IL-1β levels, and restored EPCs’ population in male mice. Taken together, these data suggest that pulmonary SOD1 may play a critical role in sex differences in ROS production and EPC’s populations in circulation and bone marrow in response to PM exposure in mice.

Studies with both human subjects and murine models have shown that PM or nickel nanoparticle exposure significantly decreases the population of circulating EPCs due to increased oxidative stress or inhibition of VEGF-mediated mobilization of EPCs from bone marrow to the circulation [11,30,32,36]. It is well documented that PM exposure leads to an increased level of oxidative stress systematically, associated with functional and structural abnormalities in multi-organ systems including the cardiovascular system [37]. Short-term exposure to a combination of indoor air dust and ozone decreased the number of EPCs (CD34^+^/KDR^+^) with increased pulmonary oxidative stress in healthy, elderly volunteers [17]. It has been shown that the lung plays a key role in PM exposure-induced oxidative stress that contributes to an impaired number and function of EPCs [12]. SOD1 is a critical regulator of intracellular redox balance [38]. In the present study, we observed that EPC populations in the circulation and bone marrow were significantly decreased in male mice with PM exposure, along with increased intracellular ROS. Thus, we hypothesized that SOD1 expression in the lungs was decreased in male mice with PM exposure. Indeed, PM exposure significantly decreased pulmonary SOD1 protein expression in male mice, but not in female mice. We also observed that Tempol, SOD mimetic, effectively attenuated PM exposure-induced intracellular ROS formation and reduction of EPC levels in blood and bone marrow in male mice. These data suggest that SOD1 in the lung plays a critical role for the differential response in ROS formation to PM exposure in male and female mice. The data from the present study also showed that Tempol treatment prevented PM exposure-induced production of pro-inflammatory cytokines IL-1β and IL-6 in male mice, indicating that the effect of Tempol on PM exposure-induced ROS production and oxidative stress may be through multiple mechanisms. Indeed, Tempol treatment has been shown to reduce oxidative damage and improve cardiac contractile function in PPAR-αKO mice [39]. Clinical studies have shown that tropical use of Tempol ameliorates dietary sodium-induced cutaneous microvascular dysfunction and prevents radiation-induced alopecia in patients without safety concerns [40,41]. However, clinical studies are needed to determine the safety and efficacy of oral administration of Tempol in patients with medical conditions associated with increased levels of systematic ROS production like air pollution and hyperlipemia.

Significant sex differences in CVD presentation, severity, and progression have been observed in animal studies and clinical research, including in the setting of hypertension, coronary artery disease, cardiomyopathy, and heart failure [42]. Pre-menopausal women are often better protected against CVDs than age-compatible males [42,43]. However, the mechanisms for the substantial sex differences in CVDs have not been well defined. Naturally, the role of sex hormones has been considered and studied extensively both experimentally and clinically. Unfortunately, the data from a large clinical study, the Heart and Estrogen/Progestin Replacement Study (HERS), has shown that female hormone replacement therapy has no benefits on the primary or secondary cardiovascular outcomes in postmenopausal women (44–79 years old) [44]. Furthermore, the randomized trial Women’s Health Initiative (WHI) in 2002 has revealed that hormone-replacement therapy increased the risk of coronary heart disease and breast cancer in women after the onset of menopause [45]. These studies suggest that estrogen-independent mechanisms may be responsible for the protection of pre-menopause women against CVDs. Our previous study has also revealed that circulating EPCs are preserved in female mice with PM exposure independent of estrogen [16].

It is well known that inflammation and oxidative stress are critical to the development and progression of CVDs. Males and females have been shown to exhibit different inflammatory reactions in the cardiovascular system [46,47]. A recent study revealed that male rats, not females, with pulmonary arterial hypertension have severe perivascular inflammation in the lungs with significant fibrosis in the wall of the small pulmonary artery and right ventricular myocardium [48]. Estrogen-independent activation of regulatory T cells and genomic factors have been shown to reduce the inflammatory response and regulate immune function in females [49,50]. It is also known that inflammatory cytokines and ROS formation are closely linked. On one hand, ROS triggers the production of pro-inflammatory cytokines by activating transcription factor nuclear factor-κB (NF-κB), while on the other, cytokines can increase ROS formation through the activation of transmembrane NADPH oxidases (NOXs) [51,52]. A significant difference in ROS production has been observed between males and females. Male rats exhibited higher ROS production and oxidative stress in vascular cells than females [53]. This is in accordance with the findings from the present study that PM exposure induced significant oxidative stress and higher levels of inflammatory cytokines (IL-1β and IL-6) in male mice as compared with females. Females have been found to have a greater antioxidant capacity than males, with higher levels of some important oxidative stress biomarkers (including plasma thiobarbituric acid-reactive substances and urinary 8-iso prostaglandin F2α) in young men than in age-matched women [54]. It was also reported that SOD activity was higher in the brain and lung of female mice than that in males, while no difference was present in the kidney or heart [55].

One of the interesting findings from the present study was that the expression of pulmonary SOD1 was selectively decreased in male mice with PM exposure. SOD1 functions as an important antioxidant enzyme to decrease ROS production. Thus, male mice with decreased SOD1 expression following PM exposure are expected to have a higher level of ROS production and oxidative stress-induced tissue damages. Amyotrophic lateral sclerosis (ALS) is a motor neuron disease that is more common in men than it is in women. It has been reported that ALS-associated SOD1 mutation leads to delayed mitochondrial dysfunction, and ROS production was significantly increased in the spinal cord of G93A-SOD1 male mice, but not females [56,57]. Pulmonary SOD1-deficiency mediated oxidative stress could be an important mechanism that contributes to sex differences in EPC population in mice with PM exposure. Further studies are needed to confirm the role of pulmonary SOD1 in PM exposure-induced ROS production and reduction of EPCs using a mouse model with pulmonary-specific overexpression of SOD1. It could also be of interest to determine if Tempol treatment could have a significant effect on EPC populations in female mice with PM exposure independent of ROS production.

## 5. Conclusions

In conclusion, we demonstrated in the present study that PM exposure significantly increased ROS production and decreased EPC populations in circulation and bone marrow in male mice, not female mice, associated with decreased expression of pulmonary SOD1. Treatment with SOD mimic Tempol effectively attenuated PM exposure-induced production of ROS and pro-inflammatory cytokines IL-1β and IL-6 in male mice, and preserved EPC populations in circulation and bone marrow.

## Data Availability

Upon reasonable request, the data are available from the corresponding author.

## References

[1] Miller M.R., Newby D.E. (2020). Air pollution and cardiovascular disease: Car sick. Cardiovasc. Res..

[2] Hamanaka R.B., Mutlu G.M. (2018). Particulate Matter Air Pollution: Effects on the Cardiovascular System. Front. Endocrinol..

[3] Meo S.A., Suraya F. (2015). Effect of environmental air pollution on cardiovascular diseases. Eur. Rev. Med. Pharm. Sci..

[4] Rajagopalan S., Al-Kindi S.G., Brook R.D. (2018). Air Pollution and Cardiovascular Disease: JACC State-of-the-Art Review. J. Am. Coll. Cardiol..

[5] Endemann D.H., Schiffrin E.L. (2004). Endothelial dysfunction. J. Am. Soc. Nephrol..

[6] Gimbrone M.J., Garcia-Cardena G. (2016). Endothelial Cell Dysfunction and the Pathobiology of Atherosclerosis. Circ. Res..

[7] Singh P., O’Toole T.E., Conklin D.J., Hill B.G., Haberzettl P. (2021). Endothelial progenitor cells as critical mediators of environmental air pollution-induced cardiovascular toxicity. Am. J. Physiol. Heart Circ. Physiol..

[8] Altabas V., Altabas K., Kirigin L. (2016). Endothelial progenitor cells (EPCs) in ageing and age-related diseases: How currently available treatment modalities affect EPC biology, atherosclerosis, and cardiovascular outcomes. Mech. Ageing. Dev..

[9] Yue Y., Wang C., Benedict C., Huang G., Truongcao M., Roy R., Cimini M., Garikipati V., Cheng Z., Koch W.J. (2020). Interleukin-10 Deficiency Alters Endothelial Progenitor Cell-Derived Exosome Reparative Effect on Myocardial Repair via Integrin-Linked Kinase Enrichment. Circ. Res..

[10] Cui Y., Xie X., Jia F., He J., Li Z., Fu M., Hao H., Liu Y., Liu J.Z., Cowan P.J. (2015). Ambient fine particulate matter induces apoptosis of endothelial progenitor cells through reactive oxygen species formation. Cell Physiol. Biochem..

[11] O’Toole T.E., Hellmann J., Wheat L., Haberzettl P., Lee J., Conklin D.J., Bhatnagar A., Pope C.R. (2010). Episodic exposure to fine particulate air pollution decreases circulating levels of endothelial progenitor cells. Circ. Res..

[12] Haberzettl P., Conklin D.J., Abplanalp W.T., Bhatnagar A., O’Toole T.E. (2018). Inhalation of Fine Particulate Matter Impairs Endothelial Progenitor Cell Function Via Pulmonary Oxidative Stress. Arter. Thromb. Vasc. Biol..

[13] Rao X., Zhong J., Brook R.D., Rajagopalan S. (2018). Effect of Particulate Matter Air Pollution on Cardiovascular Oxidative Stress Pathways. Antioxid. Redox. Signal..

[14] Kattoor A.J., Pothineni N., Palagiri D., Mehta J.L. (2017). Oxidative Stress in Atherosclerosis. Curr. Atheroscler. Rep..

[15] Sinha N., Dabla P.K. (2015). Oxidative stress and antioxidants in hypertension-a current review. Curr. Hypertens. Rev..

[16] Liu X., Xiao Y., Zhu Q., Cui Y., Hao H., Wang M., Cowan P.J., Korthuis R.J., Li G., Sun Q. (2021). Circulating Endothelial Progenitor Cells Are Preserved in Female Mice Exposed to Ambient Fine Particulate Matter Independent of Estrogen. Int. J. Mol. Sci..

[17] Jantzen K., Jensen A., Kermanizadeh A., Elholm G., Sigsgaard T., Moller P., Roursgaard M., Loft S. (2018). Inhalation of House Dust and Ozone Alters Systemic Levels of Endothelial Progenitor Cells, Oxidative Stress, and Inflammation in Elderly Subjects. Toxicol. Sci..

[18] Soberanes S., Urich D., Baker C.M., Burgess Z., Chiarella S.E., Bell E.L., Ghio A.J., De Vizcaya-Ruiz A., Liu J., Ridge K.M. (2009). Mitochondrial complex III-generated oxidants activate ASK1 and JNK to induce alveolar epithelial cell death following exposure to particulate matter air pollution. J. Biol. Chem..

[19] Fang T., Lakey P., Weber R.J., Shiraiwa M. (2019). Oxidative Potential of Particulate Matter and Generation of Reactive Oxygen Species in Epithelial Lining Fluid. Environ. Sci. Technol..

[20] Wilcox C.S. (2010). Effects of tempol and redox-cycling nitroxides in models of oxidative stress. Pharmacol. Ther..

[21] Phungphong S., Kijtawornrat A., Wattanapermpool J., Bupha-Intr T. (2020). Improvement in cardiac function of ovariectomized rats by antioxidant tempol. Free Radic. Biol. Med..

[22] Simonsen U., Christensen F.H., Buus N.H. (2009). The effect of tempol on endothelium-dependent vasodilatation and blood pressure. Pharmacol. Ther..

[23] Banday A.A., Marwaha A., Tallam L.S., Lokhandwala M.F. (2005). Tempol reduces oxidative stress, improves insulin sensitivity, decreases renal dopamine D1 receptor hyperphosphorylation, and restores D1 receptor-G-protein coupling and function in obese Zucker rats. Diabetes.

[24] Yao E.H., Fukuda N., Matsumoto T., Kobayashi N., Katakawa M., Yamamoto C., Tsunemi A., Suzuki R., Ueno T., Matsumoto K. (2007). Losartan improves the impaired function of endothelial progenitor cells in hypertension via an antioxidant effect. Hypertens. Res..

[25] Silva D., Correia T., Pereira R., Da S.R., Augusto O., Queiroz R.F. (2020). Tempol reduces inflammation and oxidative damage in cigarette smoke-exposed mice by decreasing neutrophil infiltration and activating the Nrf2 pathway. Chem. Biol. Interact..

[26] Nocun M.S., Schantz M.M. (2013). Determination of selected oxygenated polycyclic aromatic hydrocarbons (oxy-PAHs) in diesel and air particulate matter standard reference materials (SRMs). Anal. Bioanal. Chem..

[27] Hoffmann D.S., Weydert C.J., Lazartigues E., Kutschke W.J., Kienzle M.F., Leach J.E., Sharma J.A., Sharma R.V., Davisson R.L. (2008). Chronic tempol prevents hypertension, proteinuria, and poor feto-placental outcomes in BPH/5 mouse model of preeclampsia. Hypertension.

[28] Fleenor B.S., Seals D.R., Zigler M.L., Sindler A.L. (2012). Superoxide-lowering therapy with TEMPOL reverses arterial dysfunction with aging in mice. Aging Cell.

[29] Martinez G.L., Ortiz M.C., Galindo M., Sanchez J.M., Sancho-Rodriguez N., Albaladejo O.M., Rodriguez M.M., Rodriguez F. (2022). Role of heme oxygenase in the regulation of the renal hemodynamics in a model of sex dependent programmed hypertension by maternal diabetes. Am. J. Physiol. Regul. Integr. Comp. Physiol..

[30] Liberda E.N., Cuevas A.K., Qu Q., Chen L.C. (2014). The acute exposure effects of inhaled nickel nanoparticles on murine endothelial progenitor cells. Inhal. Toxicol..

[31] Zielonka J., Kalyanaraman B. (2018). Small-molecule luminescent probes for the detection of cellular oxidizing and nitrating species. Free Radic. Biol. Med..

[32] Haberzettl P., Lee J., Duggineni D., McCracken J., Bolanowski D., O’Toole T.E., Bhatnagar A., Conklin D.J. (2012). Exposure to ambient air fine particulate matter prevents VEGF-induced mobilization of endothelial progenitor cells from the bone marrow. Environ. Health Perspect..

[33] Bianconi V., Sahebkar A., Kovanen P., Bagaglia F., Ricciuti B., Calabro P., Patti G., Pirro M. (2018). Endothelial and cardiac progenitor cells for cardiovascular repair: A controversial paradigm in cell therapy. Pharmacol. Ther..

[34] Schmidt-Lucke C., Rossig L., Fichtlscherer S., Vasa M., Britten M., Kamper U., Dimmeler S., Zeiher A.M. (2005). Reduced number of circulating endothelial progenitor cells predicts future cardiovascular events: Proof of concept for the clinical importance of endogenous vascular repair. Circulation.

[35] Lin C.P., Lin F.Y., Huang P.H., Chen Y.L., Chen W.C., Chen H.Y., Huang Y.C., Liao W.L., Huang H.C., Liu P.L. (2013). Endothelial progenitor cell dysfunction in cardiovascular diseases: Role of reactive oxygen species and inflammation. Biomed. Res. Int..

[36] Niu J., Liberda E.N., Qu S., Guo X., Li X., Zhang J., Meng J., Yan B., Li N., Zhong M. (2013). The role of metal components in the cardiovascular effects of PM2.5. PLoS ONE.

[37] Hahad O., Lelieveld J., Birklein F., Lieb K., Daiber A., Munzel T. (2020). Ambient Air Pollution Increases the Risk of Cerebrovascular and Neuropsychiatric Disorders through Induction of Inflammation and Oxidative Stress. Int. J. Mol. Sci..

[38] Fukai T., Ushio-Fukai M. (2011). Superoxide dismutases: Role in redox signaling, vascular function, and diseases. Antioxid. Redox. Signal..

[39] Guellich A., Damy T., Conti M., Claes V., Samuel J.L., Pineau T., Lecarpentier Y., Coirault C. (2013). Tempol prevents cardiac oxidative damage and left ventricular dysfunction in the PPAR-alpha KO mouse. Am. J. Physiol. Heart. Circ. Physiol..

[40] Metz J.M., Smith D., Mick R., Lustig R., Mitchell J., Cherakuri M., Glatstein E., Hahn S.M. (2004). A phase I study of topical Tempol for the prevention of alopecia induced by whole brain radiotherapy. Clin. Cancer Res..

[41] Ramick M.G., Brian M.S., Matthews E.L., Patik J.C., Seals D.R., Lennon S.L., Farquhar W.B., Edwards D.G. (2019). Apocynin and Tempol ameliorate dietary sodium-induced declines in cutaneous microvascular function in salt-resistant humans. Am. J. Physiol. Heart Circ. Physiol..

[42] Regitz-Zagrosek V., Kararigas G. (2017). Mechanistic Pathways of Sex Differences in Cardiovascular Disease. Physiol. Rev..

[43] Arnold A.P., Cassis L.A., Eghbali M., Reue K., Sandberg K. (2017). Sex Hormones and Sex Chromosomes Cause Sex Differences in the Development of Cardiovascular Diseases. Arter. Thromb. Vasc. Biol..

[44] Iorga A., Cunningham C.M., Moazeni S., Ruffenach G., Umar S., Eghbali M. (2017). The protective role of estrogen and estrogen receptors in cardiovascular disease and the controversial use of estrogen therapy. Biol. Sex Differ..

[45] Lobo R.A. (2017). Hormone-replacement therapy: Current thinking. Nat. Rev. Endocrinol..

[46] Fang L., Gao X.M., Moore X.L., Kiriazis H., Su Y., Ming Z., Lim Y.L., Dart A.M., Du X.J. (2007). Differences in inflammation, MMP activation and collagen damage account for gender difference in murine cardiac rupture following myocardial infarction. J. Mol. Cell Cardiol..

[47] Janczewski A.M., Kadokami T., Lemster B., Frye C.S., McTiernan C.F., Feldman A.M. (2003). Morphological and functional changes in cardiac myocytes isolated from mice overexpressing TNF-alpha. Am. J. Physiol. Heart. Circ. Physiol..

[48] Zemskova M., Kurdyukov S., James J., McClain N., Rafikov R., Rafikova O. (2020). Sex-specific stress response and HMGB1 release in pulmonary endothelial cells. PLoS ONE.

[49] Du S., Itoh N., Askarinam S., Hill H., Arnold A.P., Voskuhl R.R. (2014). XY sex chromosome complement, compared with XX, in the CNS confers greater neurodegeneration during experimental autoimmune encephalomyelitis. Proc. Natl. Acad. Sci. USA.

[50] Tamosiuniene R., Manouvakhova O., Mesange P., Saito T., Qian J., Sanyal M., Lin Y.C., Nguyen L.P., Luria A., Tu A.B. (2018). Dominant Role for Regulatory T Cells in Protecting Females Against Pulmonary Hypertension. Circ. Res..

[51] Morgan M.J., Liu Z.G. (2011). Crosstalk of reactive oxygen species and NF-kappaB signaling. Cell. Res..

[52] Blaser H., Dostert C., Mak T.W., Brenner D. (2016). TNF and ROS Crosstalk in Inflammation. Trends Cell Biol..

[53] Barp J., Araujo A.S., Fernandes T.R., Rigatto K.V., Llesuy S., Bello-Klein A., Singal P. (2002). Myocardial antioxidant and oxidative stress changes due to sex hormones. Braz. J. Med. Biol. Res..

[54] Ide T., Tsutsui H., Ohashi N., Hayashidani S., Suematsu N., Tsuchihashi M., Tamai H., Takeshita A. (2002). Greater oxidative stress in healthy young men compared with premenopausal women. Arter. Thromb. Vasc. Biol..

[55] Chen Y., Ji L.L., Liu T.Y., Wang Z.T. (2011). Evaluation of gender-related differences in various oxidative stress enzymes in mice. Chin. J. Physiol..

[56] Cacabelos D., Ramirez-Nunez O., Granado-Serrano A.B., Torres P., Ayala V., Moiseeva V., Povedano M., Ferrer I., Pamplona R., Portero-Otin M. (2016). Early and gender-specific differences in spinal cord mitochondrial function and oxidative stress markers in a mouse model of ALS. Acta Neuropathol. Commun..

[57] Naumenko N., Pollari E., Kurronen A., Giniatullina R., Shakirzyanova A., Magga J., Koistinaho J., Giniatullin R. (2011). Gender-Specific Mechanism of Synaptic Impairment and Its Prevention by GCSF in a Mouse Model of ALS. Front. Cell. Neurosci..

